# Sex differences in resting state EEG spectral power are more prominent than menstrual cycle effects in healthy young adults

**DOI:** 10.3389/fendo.2026.1785349

**Published:** 2026-06-30

**Authors:** Angelika K. Sawicka, Aleksandra M. Zieminska, Natalia Zalewska, Adrianna Czerwińska, Katarzyna M. Michalak, Barbara Naparło, Nastaran Hamedi, Jesús S. García-Salinas, Anna B. Marcinkowska, Michal T. Kucewicz, Paweł J. Winklewski

**Affiliations:** 1 Applied Cognitive Neuroscience Lab, Department of Neurophysiology, Neuropsychology and Neuroinformatics, Medical University of Gdansk, Gdansk, Poland; 2 Brain & Mind Electrophysiology Laboratory, BioTechMed Center, Multimedia Systems Department, Faculty of Electronics, Telecommunications and Informatics, Gdansk University of Technology, Gdansk, Poland; 3 Department of Neurophysiology, Neuropsychology and Neuroinformatics, Medical University of Gdansk, Gdansk, Poland

**Keywords:** default mode network, estradiol, menstrual cycle, network oscillations, progesterone, sex differences, spectral power, testosterone

## Abstract

**Background:**

Sex hormones modulate brain function, but their specific effects on neural oscillations remain incompletely understood. This study examined associations between sex hormone concentrations and resting-state EEG spectral power in healthy young adults, comparing women with high versus low estradiol levels assessed during pre-ovulatory and menstrual cycle phases respectively, alongside sex differences between women and men.

**Methods:**

We examined resting-state EEG spectral power in 57 healthy adults (26 men, 31 women) using verified hormone measurements. Women were assessed twice across menstrual cycle phases (menstrual and pre-ovulatory), while men were assessed once. Spectral power analysis was performed across seven EEG frequency bands. Statistical comparisons of EEG group differences employed a non-parametric cluster-based permutation framework, using paired and independent-samples Wilcoxon statistics depending on the nature of the comparison. Hormone–EEG associations were assessed separately within each sex: Spearman rank correlations for males and linear mixed-effects models for females, with FDR correction.

**Results:**

Sex differences in spectral power were more prominent than differences between the investigated cycle phases, with no significant differences observed between the two female groups. Group-level comparisons revealed widespread sex differences in high-frequency bands predominantly in frontal and central-left regions. Within the male group, no hormone–EEG associations survived FDR correction. Within-female analyses revealed focal associations of estradiol with posterior high gamma power, and of progesterone with theta and high beta power at temporo-frontal and parietal sites.

**Conclusions:**

Sex hormones relate to resting-state EEG oscillations through two distinct levels of influence: long-term organizational shaping of cortical architecture, reflected in widespread between-sex differences, and acute receptor-mediated modulation, reflected in focal within-female associations. These findings underscore the importance of considering both sex and hormonal status as fundamental biological variables in electrophysiological research.

## 1 Introduction

The complex relationship between sex hormone levels and brain activity offers critical insights into various cognitive and behavioral processes, as fluctuations in steroid hormones and the distribution of their receptors play a pivotal role in the regulation of decision-making, memory, sexual and socio-sexual behaviors, aggression, neurogenesis, learning, stress responses, mood, and emotional regulation (1, 2). In women, these hormonal fluctuations are primarily driven by the hypothalamic-pituitary-gonadal (HPG) axis, which regulates the production of estrogen and progesterone across the menstrual cycle. These interactions between hormones and their receptors within specific brain regions influence the brain’s electrical activity, which is reflected in measurable changes across multiple EEG frequency bands, including delta, theta, alpha, beta, and gamma oscillations (3–5). Testosterone, acting primarily through androgen receptors densely distributed in the frontal and prefrontal cortex, is consistently associated with enhanced high-frequency beta and gamma oscillations. In contrast, ovarian hormones typically exert more regionally restricted effects (6). Literature suggests that changes in cognitive performance correlate with oscillatory activity across multiple EEG frequency bands, including theta and alpha rhythms, which have been particularly linked to memory encoding, attentional processes, and executive function (7). A common underlying mechanism may be the activation of central nervous system monoaminergic pathways, which are known to be involved in steroid feedback (2, 8). Given these influences, a measurable relationship between hormone levels and brain signals is expected to serve as a key indicator of hormone-driven neural dynamics.

Ovarian hormones have been shown to exert neuromodulatory effects, with estrogen increasing and progesterone decreasing neuronal excitability (9). The menstrual cycle consists of a follicular phase, during which estradiol is the dominant hormone and rises to its pre-ovulatory peak, and a luteal phase, in which progesterone predominates following ovulation (10, 11). Estradiol exerts positive feedback on the hypothalamic-pituitary axis, triggering an luteinizing hormone (LH) surge that induces ovulation, after which progesterone rises markedly as the corpus luteum forms. Sex hormone concentrations thus fluctuate considerably across the menstrual cycle, with estradiol and progesterone showing the most prominent cyclic change. Although testosterone is traditionally considered relatively stable in men, recent evidence shows that testosterone levels can also vary diurnally (12, 13) and seasonally (14) in both sexes, and across the menstrual cycle in women (15). Nevertheless, the magnitude of these testosterone fluctuations remains modest compared to the dramatic cyclical changes in estradiol and progesterone observed across the menstrual cycle. This contrast between a high-amplitude, multi-week hormonal cycle in women and a lower-amplitude, 24-hour rhythm in men provides an opportunity to examine both within-subject hormonal effects and between-sex differences in neural activity patterns. The relatively stable testosterone profile in men, in contrast to the cyclical fluctuations in women, may exert a tonic modulatory influence on cortical networks via androgen receptor signaling, which has been documented in the prefrontal cortex and other components of the mesocorticolimbic system (16).

EEG spectral power analysis serves as a robust tool to examine the neural correlates of brain states, as distinct frequency bands reflect specific physiological and functional processes (17). While active engagement in cognitive and emotional tasks modulates multiple rhythms, such as theta (memory and control), alpha (cortical excitability), and high-frequency beta and gamma (sensorimotor and higher-order integration), hormonal fluctuations across the menstrual cycle have also been shown to strongly influence these baseline oscillatory patterns even in the resting state (3, 18–20). For instance, resting-state alpha power is modulated by estrogen, exhibiting lower values during the primary estradiol peak in the late follicular phase (3), which suggests a direct hormonal impact on baseline cortical excitability. Conversely, beta frequency power, which is associated with enhanced alertness, typically increases during phases of low progesterone (21–23). These hormone-driven electrophysiological modulations occur within the context of broader sex differences in brain organization.

Additionally, complementary evidence from magnetoencephalography (MEG)-based source-level analysis indicates that theta activity in the right temporal cortex and the right limbic system is significantly reduced during menstruation compared to non-menstrual phases. Similarly, high-frequency gamma power in the left parietal cortex is also significantly lower during menstruation (24). These MEG findings align with and extend the EEG literature by providing enhanced spatial localization of menstrual cycle-related oscillatory changes (24).

These electrophysiological observations of hormone-related changes occur within the context of broader sex differences in brain organization, which may, in turn, be shaped by long-term hormonal exposure during development and adulthood. Evidence from neuroimaging studies suggests differences between the sexes in large-scale brain organization: males exhibit stronger functional connectivity between networks, while females show stronger intra-network connectivity within systems such as the sensorimotor, salience, auditory, and executive control networks (25). These functional patterns correspond to fundamental structural differences: women have been shown to exhibit stronger structural connectivity in the left hemisphere and higher functional connectivity (26). This combination of structural and functional evidence supports the view that the organization of the female brain favors more localized, intra-network specialization, whereas the organization of the male brain favors more diffuse integration between networks (27). The dense distribution of androgen in the frontal and prefrontal regions further suggests that these areas may be particularly sensitive to hormonal modulation of resting-state activity (6). Collectively, these findings suggest that hormone-driven modulation of EEG spectral power operates through at least complementary pathways (1): sex hormones may influence neuronal excitability by mechanisms mediated by receptors (2); these hormonal effects on local neuronal excitability can propagate through structural and functional networks, shaping the spatial distribution of spectral power across the brain cortex. Despite this body of evidence, direct comparisons of sex hormone effects using within-subject designs with verified hormone measurements remain scarce. Although numerous studies have reported EEG changes during the menstrual cycle (3, 5, 21, 22, 24), it remains unclear whether hormone-induced neural fluctuations in women are greater or smaller than the resting-state electrophysiological differences observed between women and men.

The present study aimed to address this gap by examining the relationship between sex hormone concentrations and spatial distribution of resting-state EEG spectral power in healthy young adults. Given the region-specific distribution of sex steroid receptors across the cerebral cortex - with high androgen receptor density in frontal regions and predominantly posterior estrogen receptor β (ERβ) expression - we specifically investigated the topographical patterns of these hormone-EEG associations. We employed a within-subject design for women across two specific menstrual cycle phases - the menstrual and late follicular phases - a design chosen to isolate the effects of the primary estradiol peak while minimizing the confounding influence of progesterone. Furthermore, we used between-subject comparisons with men. We hypothesized that (1): between-sex differences in spectral power would be more prominent than those originating from within-sex hormonal fluctuations observed between the investigated cycle phases, (2) testosterone would show stronger and more widespread effects compared to estradiol, (3) estradiol effects would be localized primarily to specific posterior cortical regions, and (4) strong frontal and central correlations of resting-state EEG activity with testosterone levels would reflect its role in establishing a tonic modulatory influence on prefrontal and sensorimotor networks.

## 2 Materials and methods

### 2.1 Participants

From December 2022 to November 2023, participants were enrolled through the Medical University of Gdansk’s mailing lists and advertisements on social media platforms. A total of 75 healthy young adults participated in the study, comprising 29 men and 46 women. After data pre-processing and quality control, 11 participants were excluded due to excessive EEG artifacts and incomplete data. Following hormonal verification of cycle phase, 7 additional women were excluded due to hormonal profiles inconsistent with the late follicular phase, as indicated by elevated progesterone levels and the absence of an expected estradiol rise between recording sessions. The final analysis included 57 participants (26 men and 31 women). The age of participants ranged from 20 to 35 years (M = 23.44; SD = 3.31). All participants were right-handed.

The inclusion criteria for all participants included an age range of 18–35 years and native proficiency in Polish. For women, additional requirements included a regular menstrual cycle with a length of 24 to 38 days and inter-cycle variation not exceeding 8 days, as defined by the FIGO 2018 guidelines (28). The study group was homogeneous regarding physical activity levels. All participants reported only low-to-moderate recreational exercise (≤3 times/week), with no professional or intense daily training patterns.

Exclusion criteria encompassed: (1) neurological or psychiatric conditions and use of psychiatric medications; (2) endocrine disorders, including polycystic ovary syndrome (PCOS) and endometriosis and diabetes; (3) other chronic conditions with known effects on neural activity or hormonal regulation, such as chronic inflammatory or autoimmune diseases; and (4) for women: current hormonal contraceptive use or discontinuation within 6 months prior to the study, pregnancy, postpartum period, or breastfeeding within one year before enrollment. Compliance with all inclusion and exclusion criteria was verified through a structured self-report questionnaire administered during recruitment.

Informed consent was obtained from each participant. The study was approved by the Medical University of Gdansk ethics committee (approval numbers: NKBBN/398/2021 and NKBBN/398-14/2023) in accordance with the relevant guidelines of the Declaration of Helsinki.

### 2.2 Experimental design

**Figure 1:**
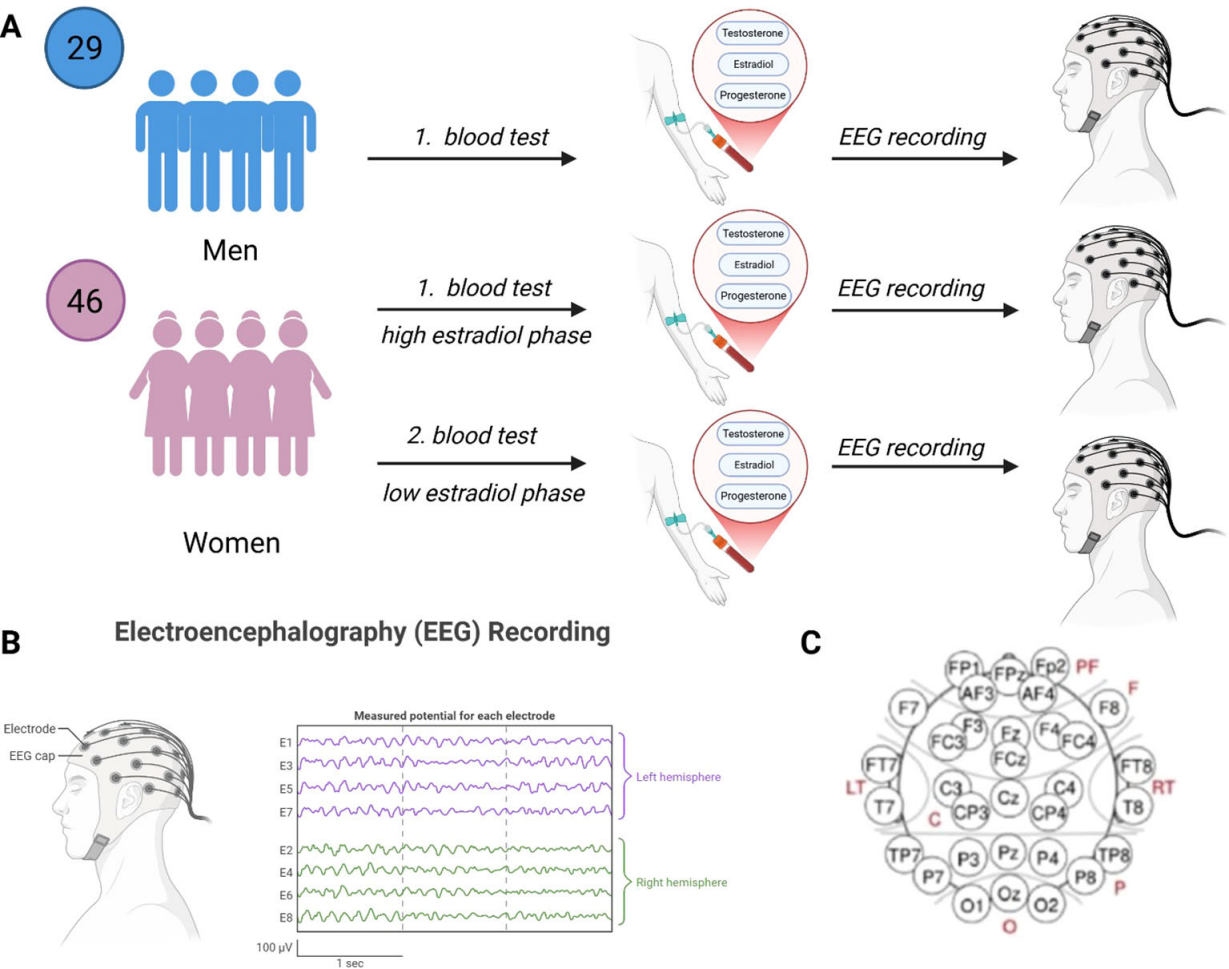
Experimental design and EEG electrode configuration. **(A)** Schematic representation of the study procedure. Men were assessed once, while women were assessed twice: in the menstrual phase and pre-ovulatory phase. **(B)** Example of pre-processed, clean EEG signals recorded from electrodes, illustrating data quality. **(C)** 32-channel electrode arrangement (International 10–20 system) used for resting-state recordings. Created with Biorender.com.

The examination procedure consisted of taking blood samples to measure sex hormone levels (estradiol, progesterone and testosterone) and performing EEG measurements. Women underwent the examination procedure during two different phases of their menstrual cycle: the menstrual phase (days 2–5 of the menstrual cycle, characterized by low estradiol and progesterone levels) and the late follicular phase (0–2 days before expected ovulation, characterized by high estradiol and low progesterone levels). Men underwent the examination once (**Figure 1A**). This approach enabled a direct comparison of neural activity in the same women across varying hormonal states (low and high estradiol levels), while providing reference data from males.

**Figure 2:**
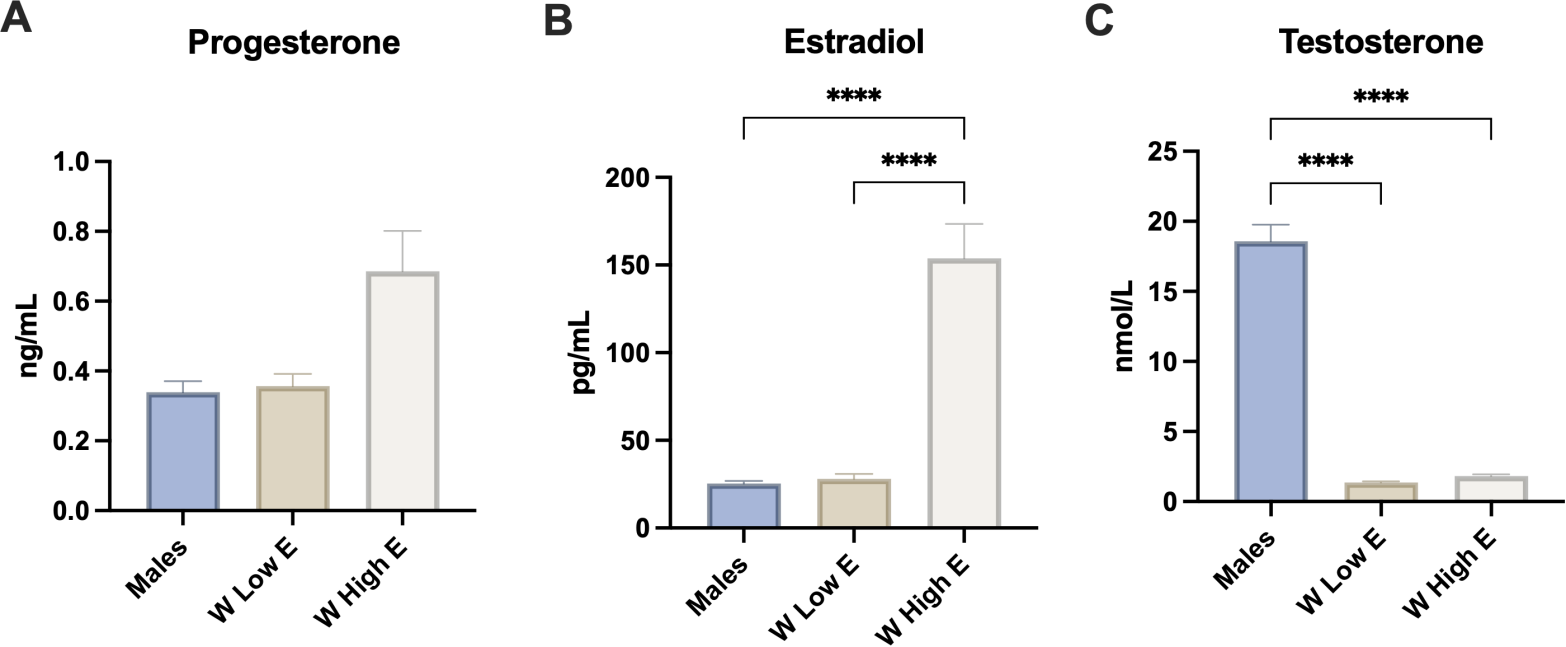
Hormone levels across study groups. **(A)** Progesterone levels (ng/mL), **(B)** estradiol levels (pg/mL), and **(C)** testosterone levels (nmol/L) in Males (n = 26), Women in the low estradiol phase (W Low E, n = 31), and Women in the high estradiol phase (W High E, n = 31). Data are presented as mean ± SEM. Between-group comparisons across all three groups were performed using the Kruskal-Wallis test with Dunn’s *post-hoc* correction. Within-group comparisons between W Low E and W High E were performed using the Wilcoxon matched-pairs signed rank test. Significant differences are indicated by **** (p < 0.0001). Progesterone levels remained within the pre-ovulatory reference range across all groups (standard reference range for follicular phase < 1.5 ng/mL).

For statistical analyses, participants were categorized into three hormone-based groups: women in the high estradiol phase (W High E, n = 31, women during the late follicular phase), women in the low estradiol phase (W Low E, n = 31, women during the menstrual phase), and group of men (Males, n = 26). The average hormone levels clearly define the established groups, as shown in **Figure 2**.

Detailed methodology for menstrual cycle verification, hormone measurement protocols, and timing procedures has been described in our previous publication (29).

### 2.3 Hormone measurements

Fasting blood samples were collected between 7:00-10:00 a.m. before each EEG session. Women provided samples during both phases of the cycle, while men were sampled only once. All samples were analyzed in ISO 15189-accredited medical diagnostic laboratory using electrochemiluminescence immunoassay (ECLIA) on a Cobas Pro analyzer (Roche Diagnostics, Basel, Switzerland) to determine estradiol, progesterone, and testosterone levels.

### 2.4 EEG recordings

EEG recordings were conducted between 8:00 a.m. and 12:00 p.m. (after blood collection) to control for circadian effects. Participants were instructed to abstain from caffeine from the evening prior to each recording session, ensuring a standard overnight washout period of at least 8 hours, in line with established EEG preparation protocols (30). EEG data were acquired using a BitBrain Versatile EEG system with 32 semi-dry electrodes positioned according to the international 10–20 system and a sampling rate of 256 Hz. Two resting-state conditions were recorded consecutively in a quiet environment, each lasting 4 minutes: (1) an eyes-open (EO) condition, in which participants fixated on a cross displayed on a monitor, and (2) an eyes-closed (EC) condition, in which participants sat quietly with eyes closed. The order of conditions was counterbalanced across participants.

### 2.5 Data analysis

EEG data pre-processing and results were performed using the EEGLAB toolbox (31) in MATLAB R2024a (MathWorks, Inc.). Prior to preprocessing, each EEG recording underwent manual quality assessment to identify poor-quality signals, excessive noise, and electrode detachment artifacts. Raw EEG data were filtered using a notch filter at 50 Hz (line noise) and a bandpass FIR filter between 0.5 and 60 Hz to reduce noise. Preliminary artifact rejection was performed semi-manually to remove large periodic noise, instances of channel contact loss, and muscle activity. A clean signal example is shown in **Figure 1B**.

Following independent component analysis (ICA), artifactual components were identified using both the ADJUST (32) and ICLabel algorithms. Components with a probability greater than 0.9 for muscle, eye, or heart artifact categories were rejected, while ambiguous cases were validated by visual inspection.

The cleaned EEG recordings were segmented into 10-second epochs. Power spectral analysis was conducted for each epoch to compute the spectral EEG power across predefined frequency bands: Delta (0.5–4 Hz), Theta (4–8 Hz), Alpha (8–12 Hz), Low Beta (12–20 Hz), High Beta (20–30 Hz), Low Gamma (30–40 Hz), and High Gamma (40–55 Hz) (33). These frequency ranges were specifically chosen for the study of cognitive functions and the underlying neural activity; the beta and gamma bands were subdivided to achieve higher spectral resolution. To account for inter-subject variability associated with individual anatomical and physiological factors, such as skull thickness and scalp conductivity, band power values were normalized into z-scores across electrodes within each frequency band. The average power in these bands was then calculated throughout the recordings to generate topographic brain maps. To ensure consistency in the analysis, only the first 110 seconds of each resting-state recording were used for all subjects.

### 2.6 Statistical analysis

Normality of the EEG spectral power was assessed using the Lilliefors test across all channels and frequency bands. Results indicated that a substantial proportion of channels did not follow a normal distribution, with normality observed in approximately 20–40% of channels in Delta, Beta, and Gamma bands, and up to ~60–70% in Theta and Alpha bands depending on the comparison. Overall, these findings suggest that the assumption of normality was not consistently met across conditions.

To identify significant differences in neural activity across the hormone-based groups, we employed a non-parametric cluster-based permutation test applied independently for each electrode and frequency band. Two distinct test statistics were employed depending on the nature of the comparison. For the within-subject comparison between women in the high estradiol phase (W High E) and women in the low estradiol phase (W Low E), paired Wilcoxon signed-rank statistics were computed at each electrode, exploiting the repeated-measures structure of the data to increase sensitivity to within-individual hormonal changes. For between-sex comparisons (Males vs. W High E; Males vs. W Low E), independent-samples Wilcoxon rank-sum statistics were used, as these groups comprise different individuals. Electrodes exceeding a predefined threshold (α = 0.05) were grouped into spatial clusters based on their physical proximity, determined from the three-dimensional electrode coordinates and a predefined neighborhood distance. For each cluster, a cluster-level statistic was calculated as the sum of the absolute z-values within the cluster. Both types of cluster-level statistics were assessed against permutation null distributions generated with 10, 000 iterations, using random sign-flipping for the paired comparison and random label shuffling for the independent comparisons, controlling the family-wise error rate at the cluster level (p < 0.05).

The between-sex comparisons were conducted independently for each menstrual cycle phase rather than within a unified linear mixed model (LMM). This phase-specific approach was chosen to characterize how the hormonal context of each phase, a testosterone dominant male environment versus either a low-estradiol or high-estradiol female environment, independently modulates the topography and magnitude of sex differences in spectral power. We acknowledge that this pairwise strategy prioritizes phase-specific biological specificity over the statistical efficiency of a unified model, and that an LMM framework would offer advantages in variance partitioning. However, given our primary aim of characterizing how each hormonal state separately relates to between-sex EEG differences, the pairwise approach was considered more appropriate for the hypotheses under investigation.

**Figure 3:**
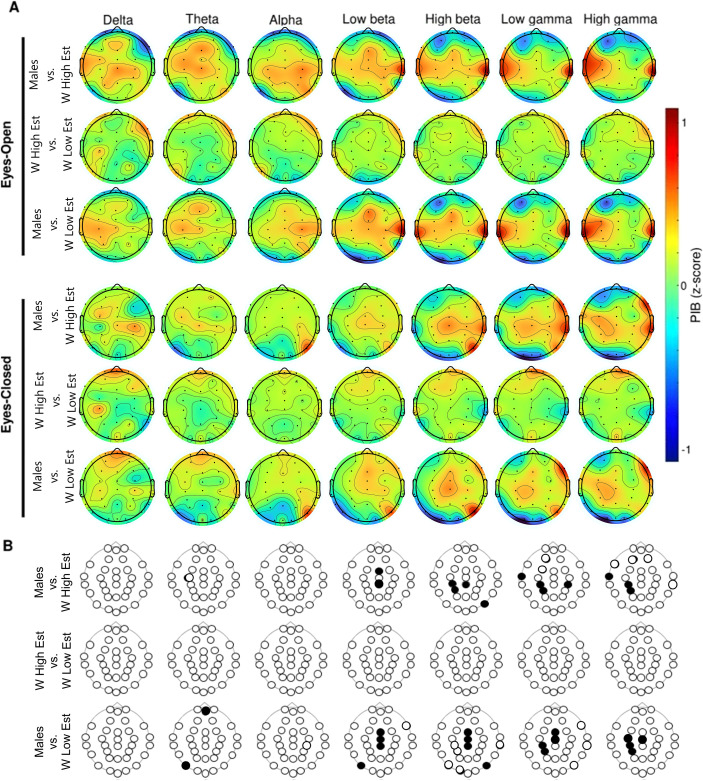
Cortical spectral power differences between hormonal groups. **(A)** Pairwise comparison of power-in-band between groups (rows), across multiple frequency bands (columns), revealed more pronounced differences between the Males and female groups. Specifically, the Males group exhibited higher power in the temporal lobes and lower power in the frontal and occipital lobes compared to the female groups. In contrast, comparisons between W Low E and W High E groups did not show relevant differences. **(B)** Statistical analyses confirmed significant differences between the Males group and both female groups, predominantly in high-frequency bands (low beta, high beta, low gamma and high gamma), distributed across frontal, central, and parietal regions with a predominance in the central-left hemisphere. Black dots indicate individual electrodes belonging to statistically significant clusters identified by cluster-based permutation testing (cluster-level p < 0.05; **Supplementary Tables S1**, **S2**).

Significant clusters were visualized as topographical maps of electrode activity for each frequency band (see **Figure 3B**), highlighting spatially contiguous regions showing reliable group differences. To evaluate whether age influenced neural activity, another cluster-based permutation regression analysis across all electrodes and frequency bands was performed. For each electrode, a linear model was fitted with age as a predictor of EEG spectral power, and the corresponding t-statistic for the age coefficient was obtained. Electrodes exceeding the significance threshold (α = 0.05) were grouped into spatial clusters based on electrode proximity. Cluster-level p-values were then obtained by comparing the observed cluster statistics against a permutation null distribution, controlling the family-wise error rate at p < 0.05.

Hormone distributions (progesterone, estradiol, and testosterone) were first assessed for normality using the Lilliefors test. All hormone variables significantly deviated from normality (p < 0.05), which informed the choice of Spearman rank correlations for the male analyses. To evaluate hormone–EEG associations while addressing both potential sex-related confounding and the non-independence of repeated observations in female participants, analyses were performed separately within the male and female groups. In the male group, Spearman rank correlations were computed between each hormone (estradiol, progesterone, testosterone) and normalized EEG power-in-band (PIB) values across electrodes and frequency bands. In the female group, low-estradiol and high-estradiol sessions were analyzed jointly rather than as independent groups, and hormone–EEG associations were assessed using linear mixed-effects models that explicitly accounted for within-subject repeated measures. For each electrode and frequency band, normalized PIB served as the dependent variable, hormone level (progesterone, estradiol, or testosterone) was included as a fixed effect, menstrual phase (low-estradiol vs. high-estradiol session) was included as a covariate, and participant identity was modeled as a random intercept to account for repeated measurements within individuals. The mixed-effects model was defined as: PIB ~ Hormone + Session + (1|Subject). Separate models were fitted for each electrode, frequency band, and hormone. For male analyses, Spearman’s correlation coefficient (ρ) was used as the effect size measure. For female repeated-measures analyses, regression coefficients (β) from the linear mixed-effects models were extracted. Because each hormone was measured in its native units (estradiol pg/mL; progesterone ng/mL; testosterone nmol/L) and has a distinct physiological range, β coefficients and their standard errors are not directly comparable across hormones. To enable visualization on a common, unit-free scale, β coefficients were standardized for the topographical maps by multiplying each coefficient by the ratio of the hormone and power-in-band standard deviations (β × SDhormone / SDPIB), expressing them in standard-deviation-per-standard-deviation units of comparable range to the Spearman coefficients used in males; unstandardized β coefficients and their standard errors are reported in the Results. To control for multiple comparisons, p-values were corrected using the Benjamini–Hochberg false discovery rate (FDR) procedure across electrodes and hormones within each frequency band, separately for male and female analyses. Associations were considered statistically significant at FDR-corrected p < 0.05.

Additionally, statistical analysis of hormone concentrations was performed separately to validate the experimental groups. Given the non-normal distribution of hormone values described above, non-parametric tests were applied throughout. Hormone levels were compared between groups using the Kruskal-Wallis test with Dunn’s *post-hoc* correction for multiple comparisons. Within-subject comparisons of estradiol and progesterone levels between the women in the low estradiol (W Low E) and high estradiol (W High E) phases were performed using the Wilcoxon matched-pairs signed-rank test. All hormonal analyses and data visualizations were conducted in GraphPad Prism (version 10.2.3 for macOS).

## 3 Results

### 3.1 Hormonal characteristics of study groups

Hormone levels across the three study groups are presented in **Figure 2**. Estradiol levels were significantly higher in the W High E group compared to W Low E (153.87 ± 108.93 pg/mL vs. 27.97 ± 16.46 pg/mL; Wilcoxon: W = 496, p < 0.0001). Progesterone levels did not differ significantly between phases (W High E: 0.69 ± 0.65 ng/mL vs. W Low E: 0.36 ± 0.19 ng/mL; Wilcoxon: W = 137; p = 0.18), nor across all three groups (Kruskal-Wallis: p = 0.25). Estradiol levels in W High E were significantly higher than in Males (25.38 ± 7.96 pg/mL; Dunn’s: p < 0.0001), while no significant difference was observed between Males and W Low E (p > 0.999). Testosterone levels in Males (18.57 ± 6.02 nmol/L) were significantly higher than in W Low E (1.35 ± 0.5 nmol/L; p < 0.0001) and W High E (1.83 ± 0.6 nmol/L; p < 0.0001), with no significant difference between female groups (p = 0.063). All values are reported as mean ± SD.

### 3.2 Sex differences vs menstrual cycle phase effects

**Figure 4:**
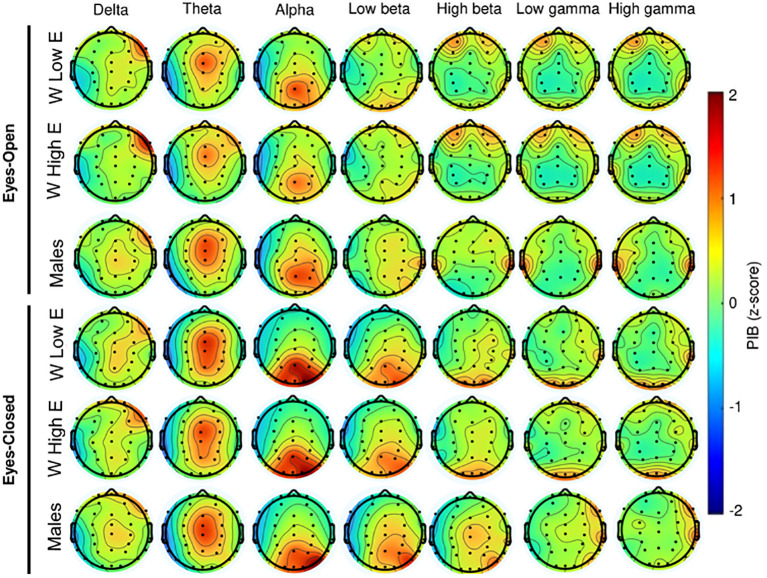
Resting-state spectral power topographies across study groups and recording conditions. Theta, alpha, and low-beta bands presented distinct activation patterns across the cortical lobes. Theta activity increased in central regions but showed a decrease in temporal areas, suggesting differentiated engagement of midline versus posterior networks. In contrast, both alpha and low-beta bands showed heightened activation in occipital–parietal regions, particularly during the eyes-closed condition, indicating consistent involvement of posterior visual and associative processing areas across groups and hormonal states. *PIB, power-in-band, expressed as z-score*.

The spectral power distributions of the three groups (Males, W Low E, and W High E) showed a similar pattern (**Figure 4**, **S1**, **S2**, **S3**). Theta band activity showed increased relative power at central electrodes and suppression in the temporal regions, especially in the eyes-closed condition. Alpha band activity was spatially concentrated in the parietal regions and extended robustly to the occipital region during the eyes-closed state, accompanied by concurrent relative suppression in the temporal region. Occipital activity was also observed in the low beta and high beta bands during the eyes-closed condition. We observed a persistent increase in power during the eyes-open condition across the high-frequency beta and gamma bands. This increase was predominantly localized to the anterior and temporal regions, forming a diffuse but consistent pattern spanning multiple high-frequency bands across all groups.

To visualize spectral power differences between groups, the power-in-band values were subtracted for each pairwise combination (**Figure 3A**). Statistical comparisons between males and each of the female groups (W High E and W Low E) revealed significant differences across multiple frequency bands (**Figures 3B**, **S4**; **Table 1**; **Supplementary Tables S1**, **S2**), with cluster-level z-statistics ranging from 3.08 to 3.93 (all p < 0.005). In contrast, the direct within-subject paired comparison between W High E and W Low E showed no significant differences after cluster-level permutation correction in either condition.

**Table 1:** Overview of resting-state EEG group differences by recording condition.

Comparison	Condition	Frequency band	Electrode clusters	Direction	Maximum statistical effect
Males vs. W Low E	EO	Low Beta, High Beta, Low Gamma, High Gamma	Frontal, Central-left	Males > W Low E	High Gamma C3: z = 3.93, p = 0.0001
Males vs. W Low E	EC	High Beta	Right Parietal	Males > W Low E	High Beta P8: z = 3.58, p = 0.0003
Males vs. W Low E	EC	Theta, Low Beta	Frontal	Males > W Low E	Theta FPz: z = 3.08, p = 0.0020
Males vs. W Low E	EC	Theta, Low Beta	Left Parietal-temporal	Males < W Low E	Theta P7: z = -3.21, p = 0.0013
Males vs. W High E	EO	Low Beta, High Beta, Low Gamma, High Gamma	Central, Left-central	Males > W High E	High Gamma C3: z = 3.79, p = 0.0002
Males vs. W High E	EC	High Beta, Low Gamma	Right Parietal, Central	Males > W High E	Low Gamma C4: z = 3.18, p = 0.0015
W High E vs. W Low E	EO/EC	All bands	Whole Brain	n.s.	No significant clusters

EO, eyes-open condition; EC, eyes-closed condition; W High E, Women in the High Estradiol phase (late follicular); W Low E, Women in the Low Estradiol phase (menstrual). Group differences were evaluated using non-parametric cluster-based permutation testing. “Direction” indicates the nature of the difference (e.g., higher power in Males), while “Maximum Statistical Effect” provides the statistical values for the individual electrode exhibiting the strongest z-statistic within each significant spatial cluster. Full statistical outputs are provided in **Supplementary Tables S1, S2**.

### 3.3 Spatial specificity and state-dependent topographical patterns

Specifically, the comparison between males and the W Low E group yielded the most widespread pattern of significant differences. In the eyes-open condition, males showed significantly higher power across the low beta (Fz, FCz, Cz), high beta (Fz, FCz, Cz), low gamma (Fz, FCz, C3, CP3), and high gamma (FC3, FCz, C3, CP3) bands, predominantly in frontal and central-left regions (largest effect: high gamma at C3, z = 3.93, p = 0.0001). During the eyes-closed condition, males showed significantly higher power in the theta band at FPz, in the low beta band at Fz, and in the high beta band at P8, while significantly lower power was observed in the theta band at P7 (z = -3.21, p = 0.0013) and in the low beta band at TP7 (z = -3.15, p = 0.0016) and P7 (z = -3.13, p = 0.0017).

A similar though slightly less widespread pattern emerged in the comparison between Males and the W High E group. In the eyes-open condition, males showed significantly higher power in the low beta (Fz, Cz), high beta (C3, CP3, Cz), low gamma (FT7, C3, CP3), and high gamma (FT7, C3, CP3) bands, distributed across central and central-left regions (largest effect: high gamma at C3, z = 3.79, p = 0.0002). During the eyes-closed condition, males showed significantly higher power in the high beta band at P8 (z = 3.07, p = 0.0022) and in the low gamma band at C4 (z = 3.18, p = 0.0015). To evaluate whether age could confound the observed hormone-related effects, we conducted cluster-based permutation tests examining the association between age and EEG spectral power across all frequency bands and eye conditions. Although several candidate clusters exceeded the initial cluster-forming threshold, none survived cluster-level permutation correction (all p > 0.05). These results indicate that age did not show a significant spatially consistent association with EEG spectral power in this dataset, suggesting that the observed spectral differences are unlikely to be driven by age-related variability.

### 3.4 Hormone-EEG correlations

In the male group (n = 26), no associations between hormone levels and EEG spectral power reached statistical significance after FDR correction in either the eyes-open or the eyes-closed condition, for any of the three hormones (all p-FDR > 0.05). In particular, testosterone showed no significant associations with EEG spectral power across electrodes or frequency bands in the male group, despite the substantial inter-individual range of testosterone levels observed (9.32–33.10 nmol/L, an approximately 3.5-fold range). The full statistical outputs across all electrodes, frequency bands, and conditions are provided in **Supplementary Data 1**.

**Figure 5:**
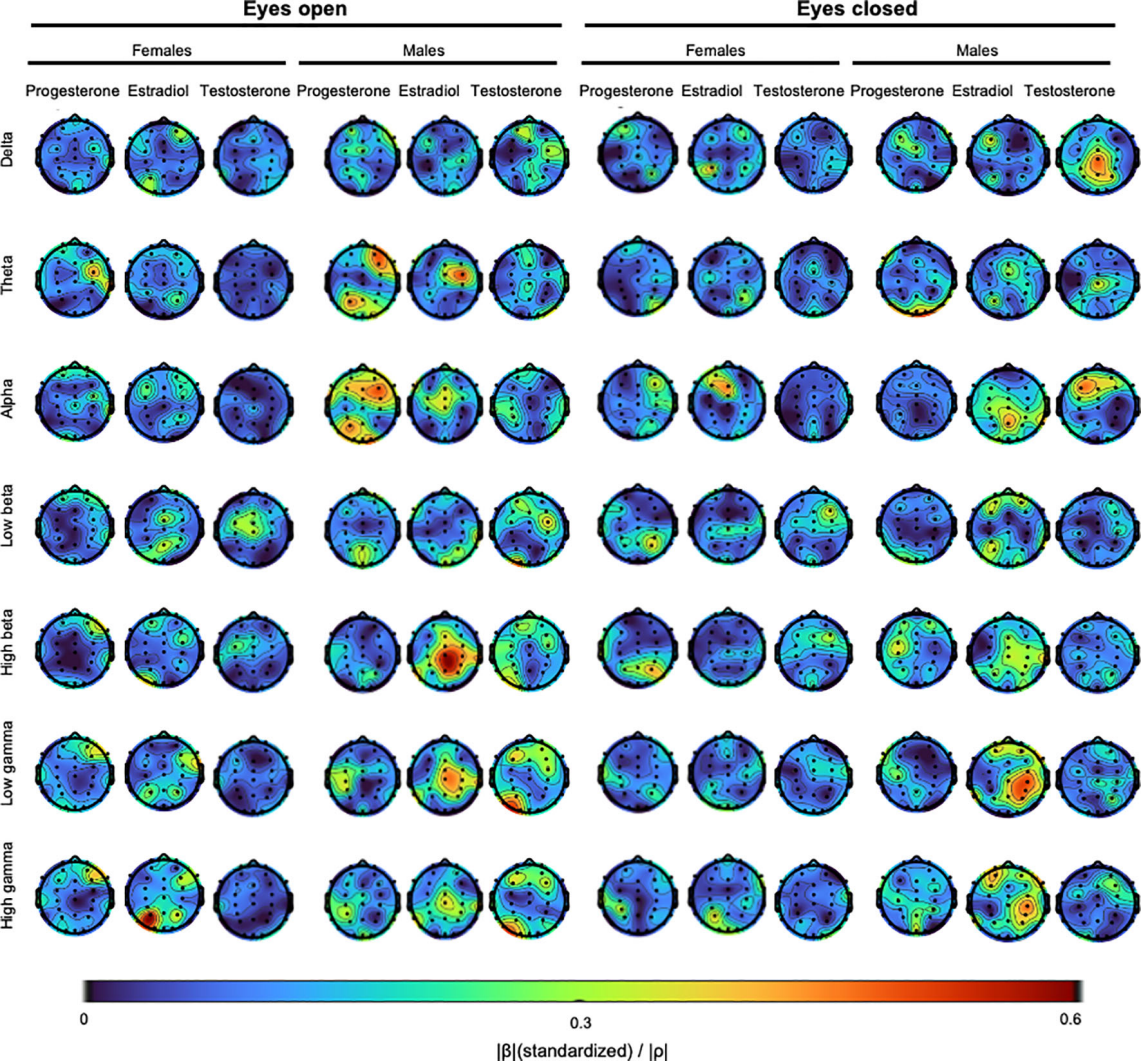
Sex-stratified hormone–power-in-band associations across frequency bands and recording conditions. Topographical maps show the magnitude of hormone–power-in-band associations for progesterone, estradiol, and testosterone across seven frequency bands (delta to high gamma) under eyes-open (left) and eyes-closed (right) conditions, separately for females and males. Female associations are shown as standardized regression coefficients (|β|) from linear mixed-effects models (standardization described in Section 2.6); male associations are shown as Spearman's rank correlation coefficients (|ρ|). Both estimators are unitless and of comparable range; a single-color scale (0-0.6) is therefore applied across all panels, with warmer colors denoting stronger associations. As |β| and |ρ| are distinct measures, intensities are not directly comparable across sexes. Maps are not thresholded by significance: after FDR correction, significant associations occurred only in females (see **Table 2**), and no male associations survived FDR correction. Full statistical outputs are provided in **Supplementary Data 1**.

In the female group (n = 31, two sessions per participant), four hormone–EEG associations remained significant after FDR correction. During the eyes-open condition, estradiol was positively associated with high gamma power at electrode P3 (β = 0.0016, SE = 0.0004, p-FDR = 0.004), and progesterone was positively associated with theta power at electrodes T8 (β = 0.88, SE = 0.19, p-FDR = 0.001) and FC4 (β = 0.58, SE = 0.15, p-FDR = 0.012). During the eyes-closed condition, progesterone was negatively associated with high beta power at electrode P4 (β = −0.51, SE = 0.14, p-FDR = 0.048). The significant progesterone associations clustered in temporo-frontal regions during eyes-open and shifted to a posterior site during eyes-closed, while the estradiol effect was spatially focal at left posterior electrode P3 (**Figure 5**). Testosterone showed no FDR-significant associations with EEG spectral power in the female group. The full linear mixed-effects outputs across all electrodes, frequency bands, conditions, and hormones are provided in **Supplementary Data 1**.

A summary of all FDR-significant findings is presented in **Table 2**.

**Table 2:** Overview of significant sex-stratified hormone–EEG associations.

Group and method	Condition	Frequency band	Hormone	Electrodes	Effect size (SE)	P (FDR)
Females (n = 31, two sessions) LMM β						
	EO	High Gamma	Estradiol	P3	β = 0.0016 (SE = 0.0004)	0.004
	EO	Theta	Progesterone	T8	β = 0.88 (SE = 0.19)	0.001
	EO	Theta	Progesterone	FC4	β = 0.58 (SE = 0.15)	0.012
	EC	High Beta	Progesterone	P4	β = -0.51 (SE = 0.14)	0.048
Males (n = 26) Spearman ρ						
	EO	All bands	All hormones	–	No significant associations after FDR	–
	EC	All bands	All hormones	–	No significant associations after FDR	–

EO, eyes-open; EC, eyes-closed. Sex-stratified hormone–EEG associations were computed separately for each sex to address the dependency between repeated female sessions and the potential sex confound. In males, single-session Spearman rank correlations were computed between each hormone and normalized EEG power across all electrodes and frequency bands. In females, linear mixed-effects models were fitted with normalized EEG power as the dependent variable, hormone level (estradiol, progesterone, or testosterone) as a fixed effect, menstrual phase as a covariate, and participant identity as a random intercept (model: PIB ~ Hormone + Session + (1|Subject)). All p-values are FDR-corrected (Benjamini–Hochberg) within each frequency band. β coefficients and their standard errors are not directly comparable across hormones because each hormone is measured in its native units (estradiol pg/mL; progesterone ng/mL; testosterone nmol/L) and has a distinct physiological range. Full statistical outputs for all electrodes, frequency bands, and hormones are provided in **Supplementary Data 1**: Full hormone EEG associations.

## 4 Discussion

### 4.1 Main findings

There are three main findings of the study: (1) sex-related differences in resting-state spectral power were more prominent and substantial than the effects of fluctuations between the investigated menstrual cycle phases (menstrual and late follicular); (2) within the male group, no FDR-corrected within-sex correlations were observed between any hormone and EEG spectral power, suggesting that the prominent between-sex differences in high-frequency activity reflect long-term organizational influences of androgen exposure rather than acute modulation by circulating hormone levels; (3) within the female group, linear mixed-effects analysis revealed anatomically and spectrally specific associations of ovarian hormones with resting-state activity - estradiol with posterior high gamma power, and progesterone with frontal-temporal theta and parietal high beta - demonstrating that circulating ovarian hormones can produce detectable modulations of baseline cortical oscillations even within a narrow phase range.

### 4.2 Sex differences vs menstrual cycle phase-related variations

Our results demonstrated that the most prominent differences in spectral power were observed between sexes, which significantly outweighed the more limited effects observed between the investigated menstrual cycle phases. A possible explanation for this pattern lies in the differing developmental contexts of hormonal exposure. The sustained, lifelong elevation of androgens in males, beginning prenatally and continuing through puberty into adulthood, may have shaped cortical architecture in ways that produce stable, widespread differences in resting-state oscillations (**Figure 3B**). In contrast, the hormonal shift in our female group was driven by a transient pre-ovulatory estradiol surge while progesterone remained at a baseline low level. This isolated estradiol peak did not produce significant group-level differences between the two female cycle phases (Section 3.2), although within-female analyses sensitive to inter-individual variation in estradiol levels did reveal a focal posterior association with high gamma power (Section 3.4).

It is worth noting that the comparison of men with the W Low E group of women revealed the widest range of significant differences, mainly in frontal and central-left regions across high-frequency bands, including low beta, high beta, and gamma. A similar, though slightly less extensive, pattern emerged when comparing men with the W High E group of women. Given that men and women in the W Low E group had comparable estradiol levels but markedly different testosterone concentrations, this pattern is consistent with testosterone being a plausible mechanistic factor underlying sex-related differences in resting-state EEG. Although, as discussed in Section 4.3, the present design cannot disentangle the specific contribution of circulating testosterone from other sex-linked factors. The slightly less pronounced pattern observed when comparing men with the W High E group may indicate that elevated estradiol levels marginally attenuate some testosterone-associated spectral differences, although this interpretation remains tentative given the overall similarity of the two comparisons.

As mentioned earlier (25, 27), neuroimaging studies have demonstrated sex differences in brain organization; males exhibit stronger inter-network functional connectivity, whereas females show enhanced intra-network connectivity. While EEG-based measures such as coherence and phase synchronization can similarly capture these connectivity differences, the present study focused on spectral power as the primary outcome measure. These structural and functional differences in brain architecture likely contribute to the observed pattern whereby between-sex differences in EEG spectral power were more prominent than the fluctuations between the menstrual and late follicular phases.

These observations suggest that sex should not be treated merely as a nuisance variable in electrophysiological studies, but as a fundamental biological factor shaping large-scale brain dynamics. While several studies report prominent EEG alterations across the menstrual cycle (22, 24, 34, 35), this discrepancy with our results likely stems from two key methodological factors. First, most previous research evaluated menstrual cycle fluctuations exclusively within female cohorts, without a male reference group. By directly contrasting these intra-individual fluctuations with between-sex differences, we demonstrate that phase-related modulations are relatively minor compared to the robust electrophysiological signatures of biological sex.

Second, earlier studies typically included the luteal phase, a period characterized by widespread γ-aminobutyric acid (GABA)ergic modulation driven by high progesterone (8, 9). Our study, however, focused exclusively on the menstrual and late follicular phases to isolate the pre-ovulatory estradiol surge. In this low-progesterone environment, the estradiol surge alone did not produce widespread between-group differences (Section 3.2), although a focal posterior association with high gamma power emerged in within-female analyses (Section 3.4, see also Section 4.3). This pattern suggests that, in the low-progesterone environment of the late follicular phase, estradiol may modulate resting-state activity in a topographically focal manner rather than through broad oscillatory shifts. Whether estradiol effects become more widespread when accompanied by elevated progesterone in the luteal phase remains an open question for future research.

### 4.3 Spatial specificity, lateralization, and state-dependent EEG patterns

Our findings revealed distinct theta-band patterns, with elevated activity over central electrodes and suppression in temporal regions, where the spatial distribution of significant electrodes appeared qualitatively more prominent in the left hemisphere. This apparent left-lateralized pattern is noteworthy given that left temporal theta oscillations have been specifically implicated in lexical-semantic retrieval during active language processing. The theta temporal suppression observed in the resting state may reflect the baseline signature of this lateralized language network in the absence of active linguistic engagement (36, 37). Conversely, the elevated theta activity observed over central electrodes reflects the generation of midline theta. This activity is primarily driven by the anterior cingulate cortex and is a hallmark of a relaxed, internalized state typical of healthy resting wakefulness (38).

While robust sex differences were primarily observed in higher frequency bands, we also detected localized theta-band differences between Males and W Low E at prefrontal (FPz) and posterior (P7) sites during the eyes-closed condition. This sensitivity of theta rhythms to neuroendocrine status warrants further investigation given testosterone’s known effects on hippocampal theta rhythms in animal models (39), and may partly explain the localized theta differences observed between males and the W Low E group in the present study. Recent human studies have demonstrated that theta oscillations in temporal regions show sensitivity to hormonal status during cognitive tasks, with women in high-estradiol phases showing enhanced theta coherence during memory encoding (3). Consistent with this evidence, within-female analyses in the present study revealed significant positive associations between progesterone and theta power at frontal and temporal electrodes during the eyes-open condition (Section 3.4), suggesting that ovarian hormones modulate resting-state theta activity even in the absence of overt cognitive engagement. Future studies employing task-based paradigms may further characterize how hormone-dependent theta modulation observed at rest translates into active cognitive states.

Although delta band activity (0.5–4 Hz) was included in our spectral analysis, minimal group differences were observed during resting wakefulness. Neither male nor female sex-stratified analyses revealed significant hormone-related associations with delta band activity after FDR correction (Section 3.4). We controlled for potential confounds of drowsiness and circadian factors (40) by conducting recordings in the morning and instructing participants to obtain sufficient sleep prior to testing. The absence of hormone-related delta effects is consistent with the primarily sleep-associated role of delta oscillations in healthy awake adults (41, 42).

Our results reveal several distinct features of how sex hormones relate to resting-state EEG oscillations in a spatially and frequency-specific manner. The most widespread effects, manifesting as group-level differences in high-frequency bands (beta and gamma), distinguished males from both female cohorts and likely reflect long-term organizational influences of androgen exposure on cortical architecture (Section 4.1, 4.3) rather than acute modulation by circulating hormone levels. By contrast, within-female analyses revealed anatomically focal associations of ovarian hormones with resting-state activity: estradiol with high gamma power at a left posterior site, and progesterone with theta and high beta power at temporo-frontal and parietal sites (Section 3.4). Notably, group-level comparisons further indicated that males showed slightly more widespread differences when compared to the W Low E group than to the W High E group, suggesting that elevated estradiol levels may marginally attenuate some sex-associated spectral differences, particularly in the high beta and gamma bands. These higher-frequency bands appear particularly sensitive to the contrast between testosterone-dominant and estrogen-dominant hormonal environments. The contrast between widespread between-sex differences in high-frequency bands and the topographically focal within-female associations is consistent with the dissociation between organizational and acute hormonal influences discussed in Section 4.3, with the localized estradiol effect aligning with the predominantly posterior distribution of ERβ receptors in the cortex (43).

The observed hemispheric asymmetries are functionally relevant. At the group level, sex differences in high-frequency activity were predominantly distributed across frontal and central-left regions (Section 3.3), consistent with the documented presence of androgen receptors across multiple cortical regions in humans and non-human primates (44, 45). Group-level differences extending into occipital and parietal regions may reflect network-level effects propagating through the dorsal visual stream connecting occipital and parietal cortices (46), consistent with androgen receptor expression in sensory cortical areas (45). These spatial patterns may contribute to well-documented sex differences in attentional control and sensorimotor processing (47).

The state-dependent organization of resting-state EEG provides important context for interpreting hormone-related findings. Group-level sex differences showed distinct topographical signatures across recording conditions: during the eyes-open state, males exhibited higher power than female groups in frontal and central regions, whereas during the eyes-closed state, group differences extended into right parietal and central regions, with additional frontal modulation (**Figure 3A**). This state-dependent shift is consistent with the well-documented reorganization of cortical dynamics between eyes-open and eyes-closed states, where reduced sensory input and enhanced posterior alpha activity engage internally oriented network configurations including the default mode network (48–50). Although our cross-sectional design does not allow us to directly test how circulating hormones interact with these state transitions, the contrast between the two conditions underscores the importance of considering behavioral context, including the engagement of sensory and attentional systems, when investigating neuroendocrine effects on resting-state EEG spectral activity. Prior neuroimaging evidence further suggests that testosterone modulates functional lateralization in a task-dependent manner, with higher testosterone levels associated with stronger right-hemisphere activation during spatial tasks (51, 52); whether such state- or task-dependent hormonal modulation generalizes to resting-state oscillatory dynamics remains an open question for future research.

Beyond these group-level state effects, within-female hormone–EEG associations in the present study emerged in both conditions but at different cortical sites (Section 3.4); whether these topographical differences reflect distinct state-specific mechanisms of hormonal modulation or stochastic variation cannot be determined from the present data and requires dedicated investigation in future studies.

### 4.4 Sex-stratified hormone–EEG associations: mechanistic implications

To determine whether circulating hormone levels continuously modulate resting-state brain activity independently of fundamental sex differences, we evaluated hormone–EEG associations separately within the male and female cohorts. Within the male group, no FDR-corrected correlations were observed between any hormone and EEG spectral power, despite a substantial inter-individual range in testosterone concentrations (9.32–33.10 nmol/L, an approximately 3.5-fold range). This diverges from our *a priori* hypothesis (H4), which proposed that circulating testosterone tonically modulates frontal and central resting-state networks. The absence of within-sex correlations, despite a substantial within-sex range, suggests that the robust between-sex differences in high-frequency activity (Section 3.3) likely reflect long-term organizational effects of developmental androgen exposure on cortical architecture, rather than acute modulation by circulating levels (52, 53). Although the profound, ten-fold inter-sex contrast in testosterone (Males: 18.57 ± 6.02 nmol/L vs. females: ~1.3–1.8 nmol/L) perfectly aligns with the observed group-level EEG differences, our cross-sectional design cannot disentangle the specific acute contribution of circulating testosterone from inextricably linked sex factors, including sex chromosomes and prenatal organizational effects. Directly isolating the causal role of circulating testosterone in shaping resting-state spectral power requires experimental manipulation or cohorts with overlapping inter-sex hormone concentrations.

In the female group, the linear mixed-effects analysis revealed a highly focal positive association between estradiol and high gamma power at the left posterior electrode P3 during the eyes-open condition (β = 0.0016, SE = 0.0004, p-FDR = 0.004). This finding directly supports our *a priori* hypothesis (H3) regarding the posterior localization of estradiol effects and aligns with the predominantly posterior distribution of ERβ receptors in the human cortex (43). Because high gamma oscillations are primarily generated by fast-spiking parvalbumin-positive interneurons and reflect the local excitation/inhibition balance (54), the present result indicates that estradiol finely tunes resting-state local cortical excitability in posterior regions, even in the absence of explicit task engagement. This observation is consistent with prior evidence that estradiol modulates excitability in the visual and somatosensory cortices (55, 56), extending those findings into the resting-state oscillatory domain. Importantly, the extreme spatial focality of this FDR-significant effect, restricted to a single posterior site, stands in stark contrast to the widespread inter-sex differences. This localized signature suggests that ERβ-mediated estrogen action at rest is anatomically constrained, exerting precise, focal modulations on baseline cortical rhythms rather than driving broad network reorganization.

Three additional associations emerged for progesterone, although these were not predicted by our *a priori* hypotheses and should therefore be regarded as exploratory. During the eyes-open condition, progesterone was positively associated with theta power at right temporal electrode T8 (β = 0.88, SE = 0.19, p-FDR = 0.001) and at right frontal electrode FC4 (β = 0.58, SE = 0.15, p-FDR = 0.012). During the eyes-closed condition, progesterone was negatively associated with high beta power at right parietal electrode P4 (β = −0.51, SE = 0.14, p-FDR = 0.048). The frontal-temporal theta associations are consistent with prior work demonstrating that progesterone modulates theta oscillations in frontal-parietal networks (4), while the negative association in high beta extends earlier reports linking lower progesterone phases to increased beta activity (21). At a mechanistic level, progesterone and its metabolite allopregnanolone act as positive allosteric modulators of GABA-A receptors, shifting the cortical excitation/inhibition balance toward inhibition (8). This mechanism is consistent with the directionality observed here: minor elevations in baseline progesterone were associated with enhanced theta, a slower rhythm typically reflecting reduced state-dependent cortical arousal, and suppressed high-frequency beta activity. Notably, these localized associations emerged despite the inherently narrow within-cycle range of progesterone in the present cohort (~0.4–0.7 ng/mL), which deliberately excluded the luteal phase. The robustness of these focal effects under restricted baseline conditions suggests that the resting brain is sensitive to even modest physiological variations in progesterone, although replication in cohorts spanning the full menstrual cycle is warranted.

Taken together, these sex-stratified findings reveal a clear dissociation between organizational and acute hormonal influences on resting-state EEG. The most prominent between-sex differences in high-frequency activity (Section 3.3) emerged in the absence of within-sex testosterone correlations, indicating that these differences likely reflect long-term developmental shaping of cortical architecture rather than continuous modulation by current hormone levels. Conversely, the within-female associations - estradiol with posterior high gamma, and progesterone with frontal-temporal theta and parietal high beta - demonstrate that circulating ovarian hormones can produce detectable, anatomically and spectrally specific modulations of baseline cortical activity, even at modest physiological concentrations and within the narrow phase range examined here. The contrast between widespread, organizational sex effects and focal, receptor-specific within-female effects suggests that resting-state EEG simultaneously indexes two distinct levels of hormonal influence: stable, developmental scaffolding and dynamic, receptor-mediated tuning. This conceptual distinction has implications for how hormone–brain relationships should be modeled in future electrophysiological work, particularly the recognition that pooled cross-sex correlations may conflate organizational sex differences with acute hormone-driven modulation, potentially obscuring associations that emerge only within a single sex.

### 4.5 Clinical implications

The present findings have several implications for both research methodology and clinical practice. First, these results underscore the importance of considering biological sex and specific hormonal states in cognitive neuroscience research. Our data suggest that failing to account for biological sex can introduce substantial unexplained variance into electrophysiological baseline measures, while the hormonal context of specific menstrual cycle phases should be considered when designing and interpreting electrophysiological studies in female participants. Together, accounting for these fundamental biological variables is necessary to prevent the masking of true experimental effects (57). Second, understanding baseline hormone-EEG relationships in naturally cycling women provides an essential reference for evaluating how exogenous hormones - whether in contraceptives or hormone replacement therapy - alter brain function. Recent studies demonstrate that hormonal contraceptive use is associated with altered brain structure and resting-state connectivity (58), emphasizing the clinical relevance of characterizing endogenous hormone effects.

Finally, the sex-specific patterns we observed have implications for personalized medicine approaches in psychiatry and neurology. Understanding the neural mechanisms underlying sex differences in neuropsychiatric disorders - including hormone-mediated modulation of brain oscillations - may inform more targeted therapeutic interventions (59).

### 4.6 Limitations

While this study provided valuable insights, several limitations should be acknowledged. First, our sample consisted exclusively of healthy young adults (18–35 years), limiting generalizability to other age groups, particularly older women who are perimenopausal or postmenopausal, where different hormone-brain relationships may emerge (60, 61). Relatedly, although the restricted age range of our sample and a separate cluster-based permutation regression, which revealed no significant associations between age and EEG spectral power, suggest that age-related variability is unlikely to have substantially confounded the primary findings, age was not formally included as a covariate in the between-group analyses. Future studies would benefit from statistical models that explicitly incorporate age as a covariate to partition its variance from sex- and hormone-related effects. Furthermore, although we excluded women currently using hormonal contraceptives or those who discontinued use less than 6 months prior, we did not control for duration of previous contraceptive use, which may have long-term effects on brain structure and function (62–64). Another consideration is that our study focused specifically on the menstrual and late follicular phases to examine the effects of the pre-ovulatory estradiol peak. While this approach minimized the confounding influence of high progesterone, we acknowledge that excluding the luteal phase limits our ability to draw conclusions about the entire menstrual cycle. Future studies including the luteal phase would undoubtedly enrich the current findings by accounting for the potent neuromodulatory effects of progesterone. Furthermore, cycle phase classification relied on calendar-based methods combined with direct hormonal measurements of estradiol and progesterone, without LH surge testing, which may have introduced some variability in phase classification given the well-documented inter-individual variability in estradiol levels across the late follicular phase (65, 66). Additionally, testosterone levels exhibit seasonal fluctuations, with peaks in autumn and winter and a nadir in summer, and differences between monthly means of up to 31% have been reported (67). As recruitment in the present study spanned December 2022 to November 2023, covering all seasons, this variability could not be controlled for. Seasonal variation in testosterone therefore represents a potential source of variability in group-level estimates that should be considered when interpreting our findings. Additionally, the sample of male participants (n = 26) may have limited statistical power to detect within-sex testosterone correlations with EEG spectral power. While the substantial inter-individual range of testosterone levels observed in males (9.32–33.10 nmol/L, an approximately 3.5-fold range) provided sufficient variability for correlation analysis, future studies with larger male cohorts would be better positioned to detect potential within-sex hormonal effects that may have been below the detection threshold here.

Despite these limitations, the study design, which combines direct hormonal measurements with repeated EEG recordings in women across two contrasting hormonal states, provides a robust framework for isolating the modulatory influence of sex hormones on the EEG brain activity. Future studies will extend this approach by incorporating the luteal phase, repeated assessments in males, a broader age range, control for prior hormonal contraceptive use, assessment of seasonal variability in testosterone, and additional hormonal markers and LH-verified cycle phase classification. Future studies should also extend these findings by examining EEG spectral power during cognitive tasks to determine how the resting-state hormonal modulation observed here translates into cognitive performance.

## 5 Conclusions

This study demonstrates that sex hormones relate to resting-state EEG oscillations in a spatially and frequency-specific manner, with sex differences being more prominent than variations between the menstrual and late follicular phases. Sex-stratified analyses revealed that the prominent between-sex differences in high-frequency activity emerged in the absence of within-sex testosterone correlations, suggesting that these differences likely reflect long-term organizational influences of androgen exposure on cortical architecture rather than acute modulation by circulating hormone levels. By contrast, within-female analyses identified anatomically focal associations of estradiol with posterior high gamma power, and of progesterone with frontal-temporal theta and parietal high beta power, indicating that variation in circulating ovarian hormones tracks detectable modulations of baseline cortical oscillations even within a narrow phase range.

This dissociation highlights two distinct levels of hormonal influence on cortical activity: long-term developmental shaping and acute receptor-mediated modulation, and suggests that pooled cross-sex correlations may obscure associations that emerge only within a single sex. These findings underscore the importance of considering hormonal status and sex as fundamental biological variables in neuroscience research. The distinct neural signatures associated with different sex hormones have implications for optimizing personalized treatment approaches and designing more rigorous cognitive neuroscience studies that account for endocrine variability.

## Data Availability

The raw data supporting the conclusions of this article will be made available by the authors, without undue reservation.

## Supplementary material

Supplementary Table 1 Summary of significant difference p-values found between Males and W High E.

Supplementary Table 2 Summary of significant difference p-values found between Males and W Low E.

Supplementary Figure 1 Detailed comparison of band-limited EEG power between female groups across eyes-closed and eyes-open conditions.

Supplementary Figure 2 Detailed comparison of band-limited EEG power between Males and W High E groups across eyes-closed and eyes-open conditions.

Supplementary Figure 3 Detailed comparison of band-limited EEG power between Males and W Low E groups across eyes-closed and eyes-open conditions.

Supplementary Figure 4 Detailed power-in-band comparison for electrodes showing statistically significant differences between groups (**Figure 3B**).

Supplementary Data Sheet 1 Full sex-stratified hormone - EEG association results across all electrodes (32), frequency bands (7), hormones (estradiol, progesterone, testosterone), and recording conditions (eyes-open, eyes-closed). Male results report Spearman’s rank correlation coefficients (ρ); female results report regression coefficients (β) from linear mixed-effects models. Both uncorrected and FDR-corrected p-values are provided.
