# Supplementary material for: Sex differences in resting state EEG spectral power are more prominent than menstrual cycle effects in healthy young adults
Source: Front Endocrinol (Lausanne). 2026 Jun 30;17:1785349. doi: 10.3389/fendo.2026.1785349 (PMC13364567; doi:10.3389/fendo.2026.1785349)
Supplement: Supplementary Table 1 — Summary of significant difference p-values found between Males and W High E. [file SupplementaryFile1.docx]

Supplementary Material

# Supplementary Tables

**Table S1. Summary of significant difference p-values found between Males and W High E.**

| **State** | **Band** | **z-value** | **p-value** | **Channel** |
| --- | --- | --- | --- | --- |
| Closed | HBeta | 3.0682 | 0.0022 | P8 |
| Closed | LGamma | 3.1803 | 0.0015 | C4 |
| Open | LBeta | 3.1162 | 0.0018 | FZ |
| Open | LBeta | 3.1162 | 0.0018 | CZ |
| Open | HBeta | 3.2284 | 0.0012 | C3 |
| Open | HBeta | 3.4046 | 0.0007 | CP3 |
| Open | HBeta | 3.3726 | 0.0007 | CZ |
| Open | LGamma | 3.2604 | 0.0011 | FT7 |
| Open | LGamma | 3.2604 | 0.0011 | C3 |
| Open | LGamma | 3.2604 | 0.0011 | CP3 |
| Open | HGamma | 3.4046 | 0.0007 | FT7 |
| Open | HGamma | 3.7891 | 0.0002 | C3 |

Notice that FT7 and CP3 channels appear across multiple frequency bands during the eyes-open condition. *Abbreviations*: State – EEG recording condition (Open - eyes-open; Closed – eyes-closed); Band - EEG frequency band; z-value - cluster-level z-statistic; p-value - cluster-level p-value obtained from cluster-based permutation testing (10,000 permutations); Channel - EEG electrode location; W High E - Women in the High Estradiol phase

# Table S2.  Summary of significant difference p-values found between Males and W Low E.

| **State** | **Band** | **z-value** | **p-value** | **Channel** |
| --- | --- | --- | --- | --- |
| Closed | Theta | 3.0842 | 0.0020 | FPZ |
| Closed | Theta | -3.2124 | 0.0013 | P7 |
| Closed | LBeta | 3.4206 | 0.0006 | FZ |
| Closed | LBeta | -3.1483 | 0.0016 | TP7 |
| Closed | LBeta | -3.1322 | 0.0017 | P7 |
| Closed | HBeta | 3.5809 | 0.0003 | P8 |
| Open | LBeta | 3.7250 | 0.0002 | FZ |
| Open | LBeta | 3.2284 | 0.0012 | FCZ |
| Open | LBeta | 3.3085 | 0.0009 | CZ |
| Open | HBeta | 3.2925 | 0.0010 | FZ |
| Open | HBeta | 3.5168 | 0.0004 | FCZ |
| Open | HBeta | 3.1963 | 0.0014 | CZ |
| Open | LGamma | 3.2284 | 0.0012 | FZ |
| Open | LGamma | 3.4206 | 0.0006 | FCZ |
| Open | LGamma | 3.3566 | 0.0008 | C3 |
| Open | LGamma | 3.1162 | 0.0018 | CP3 |
| Open | HGamma | 3.1162 | 0.0018 | FC3 |
| Open | HGamma | 3.4687 | 0.0005 | FCZ |
| Open | HGamma | 3.9333 | 0.0001 | C3 |
| Open | HGamma | 3.6610 | 0.0003 | CP3 |

Notice that Fz appears in both conditions and across multiple frequency bands. Moreover, during the eyes-open condition, FCz, C3 and CP3 channels appear across multiple bands. *Abbreviations*: State - EEG recording condition (Open – eyes-open; Closed – eyes-closed); Band – EEG frequency band; z-value - cluster-level z-statistic; p-value - cluster-level p-value obtained from cluster-based permutation testing (10,000 permutations); Channel - EEG electrode location; W Low E - Women in the Low Estradiol phase. ρ
