# Supplementary material for: Sex differences in resting state EEG spectral power are more prominent than menstrual cycle effects in healthy young adults
Source: Front Endocrinol (Lausanne). 2026 Jun 30;17:1785349. doi: 10.3389/fendo.2026.1785349 (PMC13364567; doi:10.3389/fendo.2026.1785349)
Supplement: Supplementary Table 2 — Summary of significant difference p-values found between Males and W Low E. [file SupplementaryFile2.docx]

Supplementary Material

# Supplementary Figures Captions

**Figure S1. Detailed comparison of band-limited EEG power between female groups across eyes-closed and eyes-open conditions.** Across frequency bands, spatial variability is observed, with both positive and negative values depending on scalp location. In the delta and theta bands, both conditions exhibit mixed deviations without a consistent directional pattern; however, in the eyes-closed condition the spatial distribution is more structured, whereas in the eyes-open condition it appears more distributed. In the alpha and beta bands, an anterior–posterior gradient is evident in both conditions, with predominantly negative values in frontal and central regions and positive values in parietal and occipital areas. This gradient is more pronounced in the eyes-closed condition and becomes attenuated in the eyes-open state. Gamma-band activity displays heterogeneous fluctuations across the scalp in both conditions, with negative values more frequently observed in anterior regions and positive values in posterior regions. Compared to eyes-closed recordings, the eyes-open condition shows a more balanced and less spatially organized distribution. Error bars represent variability across participants (±SEM).

**Figure S2. Detailed comparison of band-limited EEG power between Males and W High E groups across eyes-closed and eyes-open conditions.** Across both conditions, alpha and low-beta bands demonstrate a clear anterior–posterior differentiation, with reduced power in frontal regions and increased values in parietal and occipital electrodes, more pronounced during eyes-closed recordings. Theta and delta bands exhibit region-specific variability without a consistent global pattern, although localized increases are visible in central and posterior regions, particularly in the eyes-open condition. Higher-frequency activity (high-beta and gamma bands) shows differentiated modulation across scalp regions, with mixed positive and negative deviations depending on electrode location. In the eyes-closed condition, these patterns are more spatially structured, whereas in the eyes-open condition they appear more distributed and less topographically constrained. Error bars represent variability across participants (±SEM).

**Figure S3. Detailed comparison of band-limited EEG power between Males and W Low E groups across eyes-closed and eyes-open conditions.** In the eyes-closed condition, alpha power shows a clear increase in posterior regions (parietal–occipital electrodes), with consistently higher values in the Males group. This effect is spatially selective and not prominent in frontal areas. Beta bands (low- and high-beta) exhibit partially overlapping increases in centro-parietal regions, with more heterogeneous patterns than alpha and localized reductions in frontal and temporal sites. Gamma activity is more focal, with pronounced increases in central electrodes (particularly Cz) and mixed effects across other regions. Delta and theta bands display variable, region-dependent differences across frontal, temporal, and central areas, without a consistent global trend.In the eyes-open condition, effects are generally attenuated and less spatially coherent. Posterior alpha enhancement persists but is reduced, while beta and gamma bands retain localized increases, mainly in central and parietal regions. Error bars represent variability across participants (±SEM).

**Figure S4. Detailed power-in-band comparison for electrodes showing statistically significant differences between groups (Fig 4B).** In the eyes-closed condition, differences are observed primarily in beta and gamma frequency ranges. The Males group generally exhibits higher power values at frontal and central electrodes, while lower power is observed at posterior electrodes (P7, TP7) in the theta and low beta bands. Theta band differences were also observed during the eyes-closed condition, with males showing higher power at prefrontal (FPz) and lower power at posterior (P7) electrodes. In the eyes-open condition, a larger number of significant effects is observed, predominantly within beta and gamma bands. The Males group shows higher power across frontal and central-left electrodes, with more pronounced dispersion of values compared to the eyes-closed condition. The differences are primarily expressed as shifts in distribution (median and spread), rather than uniform changes across entire frequency bands. Boxplots represent median, interquartile range, and outliers, illustrating both central tendency and inter-individual variability.
