# Supplementary figures and images for: Sex differences in resting state EEG spectral power are more prominent than menstrual cycle effects in healthy young adults
Source: Front Endocrinol (Lausanne). 2026 Jun 30;17:1785349. doi: 10.3389/fendo.2026.1785349 (PMC13364567; doi:10.3389/fendo.2026.1785349)

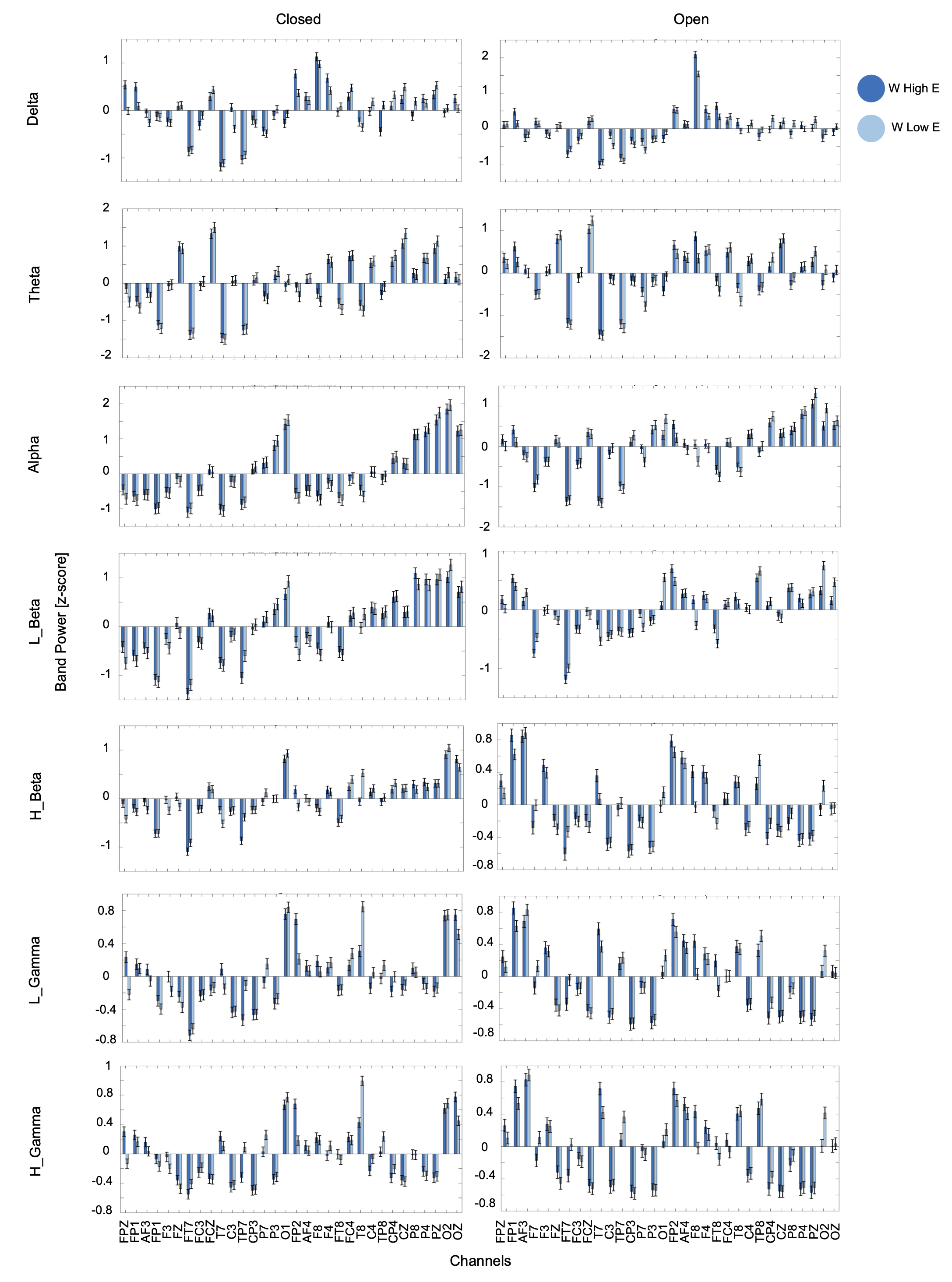

Supplement: Supplementary Figure 1 — Detailed comparison of band-limited EEG power between female groups across eyes-closed and eyes-open conditions. [file Image1.tiff]

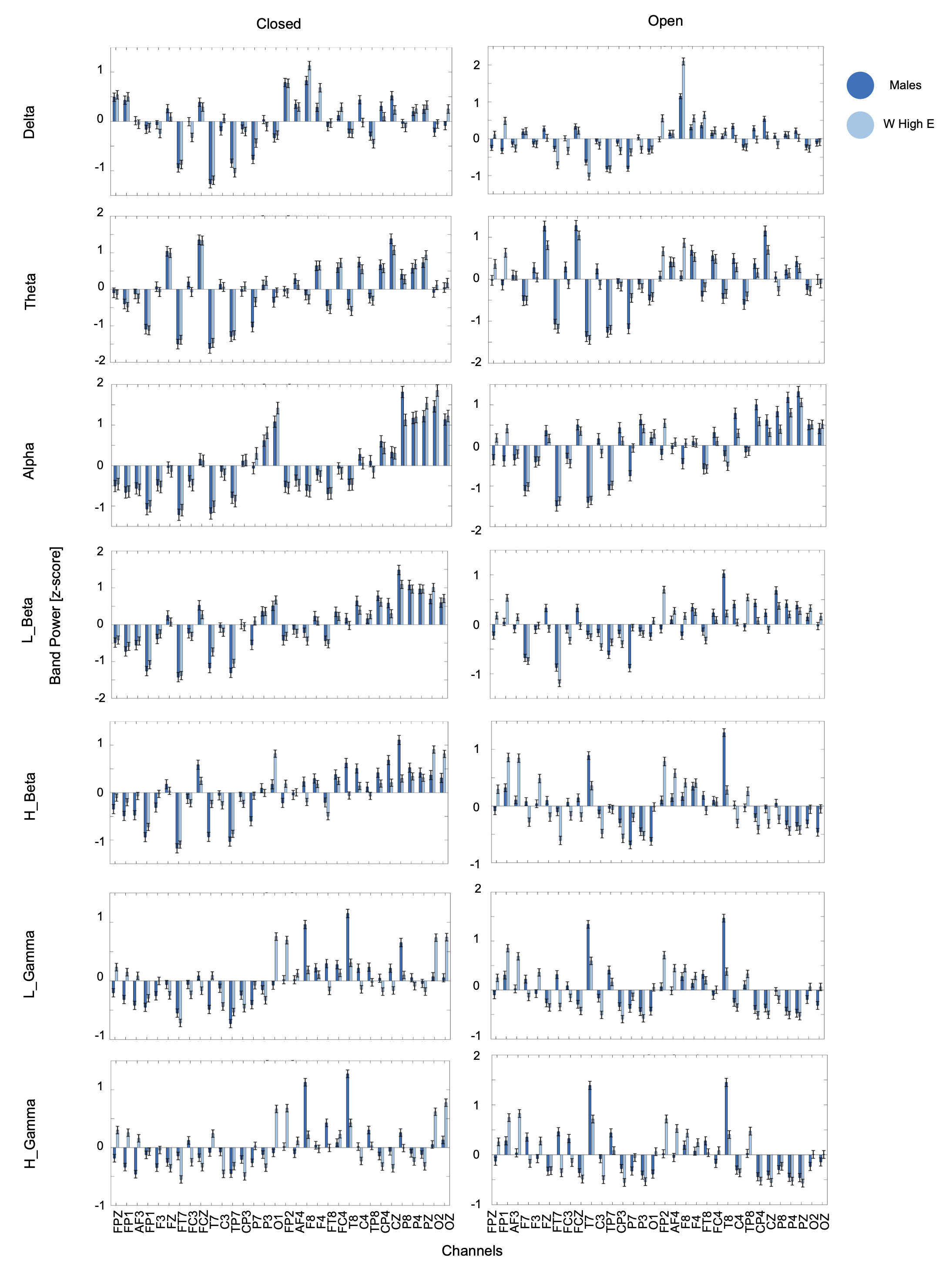

Supplement: Supplementary Figure 2 — Detailed comparison of band-limited EEG power between Males and W High E groups across eyes-closed and eyes-open conditions. [file Image2.tiff]

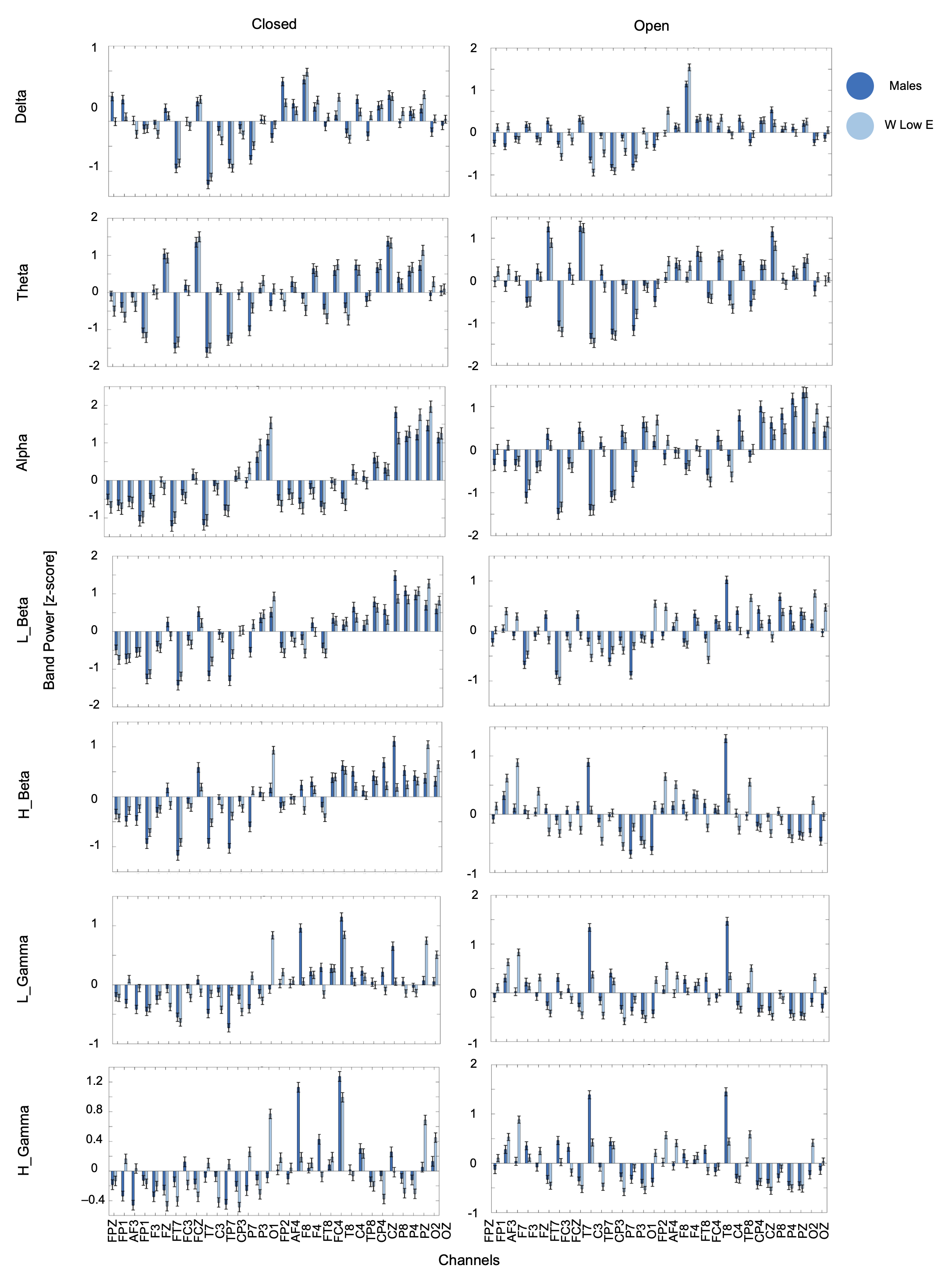

Supplement: Supplementary Figure 3 — Detailed comparison of band-limited EEG power between Males and W Low E groups across eyes-closed and eyes-open conditions. [file Image3.tiff]

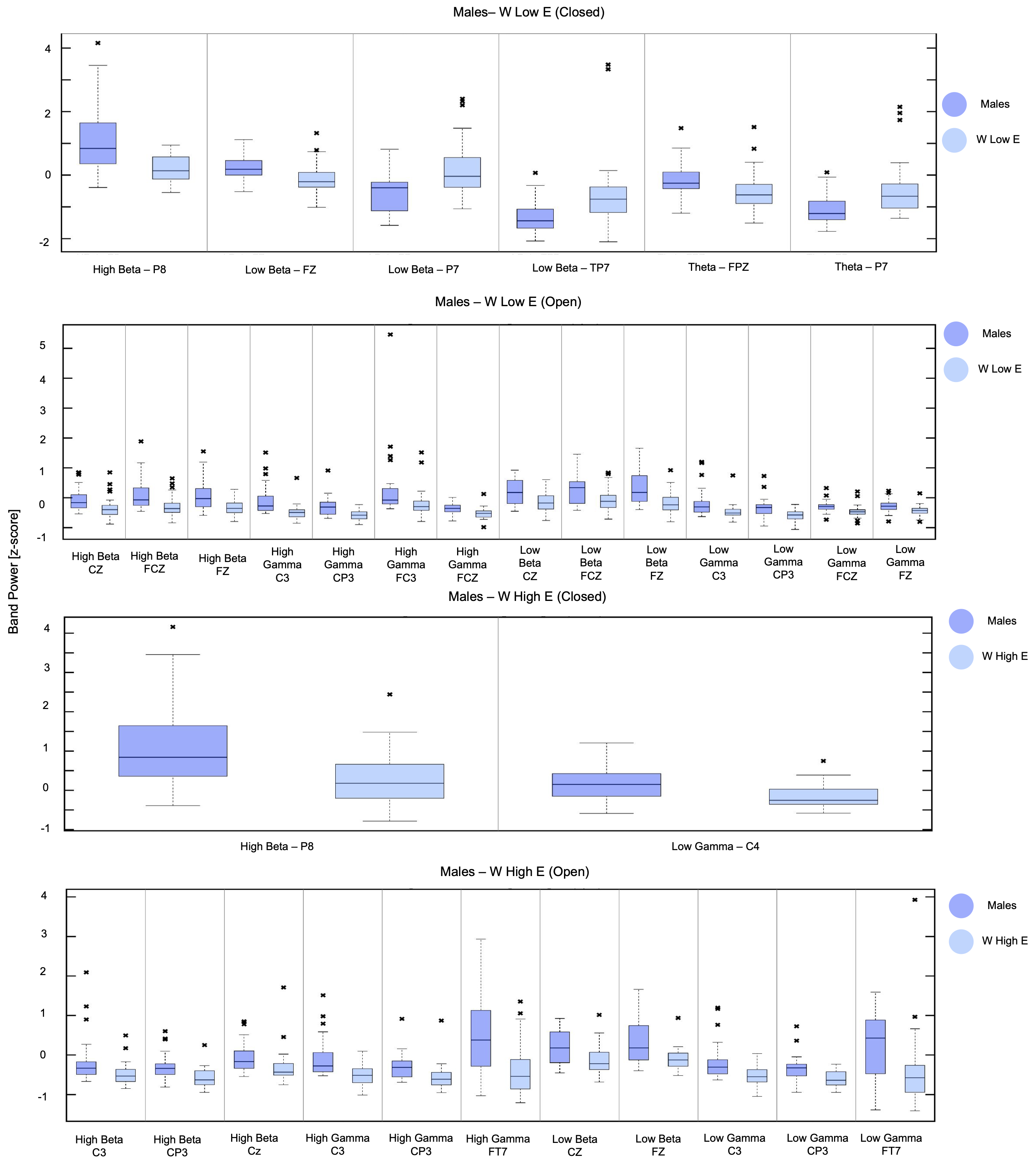

Supplement: Supplementary Figure 4 — Detailed power-in-band comparison for electrodes showing statistically significant differences between groups (Figure 3B). [file Image4.tiff]
